# Predation via motion parallax in one of two gleaning insects

**DOI:** 10.1242/jeb.251710

**Published:** 2026-03-18

**Authors:** Sergio Rossoni, Mary E. Sumner, Doekele G. Stavenga, Samuel T. Fabian, Jack A. Supple, Paloma T. Gonzalez-Bellido

**Affiliations:** ^1^ Department of Zoology, University of Cambridge, Cambridge CB2 3EJ, UK; ^2^ Department of Physiology, Development and Neuroscience, University of Cambridge, Cambridge CB3 2EG, UK; ^3^ Department of Ecology, Evolution and Behaviour, University of Minnesota, St Paul, MN 55108, USA; ^4^ Groningen Institute for Evolutionary Life Sciences, University of Groningen, Groningen 9747 AG, The Netherlands

**Keywords:** Robber fly, Damselfly, Peering, Depth perception, Active vision, Optical trade-off

## Abstract

A predator's survival is highly dependent on correctly deciding whether to attack potential prey. Pursuit predators, for example, can estimate the size of a moving target from the ratio between its angular speed and size. Such heuristic rules are not available, however, when ambushing stationary prey. Here, we investigated how pixie robber flies (*Psilonyx annulatus*) and damselflies (*Ischnura posita*) hunt stationary prey using different sensory strategies, relating to their marked differences in eye morphology. We show that pixie robber flies assess prey using whole-body translational movements. During this assessment, the prey is outside the pixie robber fly's stereopsis range, yet attacks are launched from a distance dictated by absolute, not angular, prey size. These findings suggest that pixie robber flies use motion parallax to infer three-dimensional cues, such as prey distance and/or size, before attacking. Motion parallax may be particularly suitable for pixie robber flies as they hunt in cluttered, low-lighting conditions and have a small size, making it difficult for potential prey to detect their movement, even in close proximity. Damselflies probably rely on alternative processes to assess prey, as translational movements are absent in the assessment phase.

## Teaser

**Highlighted Article:** Pixie robber flies assess the size/distance of stationary prey using motion parallax before attacking, while damselflies probably rely on binocular vision for assessing the same type of prey.

## INTRODUCTION

A predator's success is often dependent on its ability to estimate the distance and size of potential prey. The strategy by which predatory animals gather such information is highly variable between species. Pursuit predators that chase fleeing prey may favour speed of response over accuracy and thus employ a simple ratio between perceived prey speed and size to determine attack probability; closer prey appear to be larger and to move faster, compared with prey farther away. Indeed, the ratio between the prey's angular speed and its angular size predicts the likelihood of an attack in killer flies (Wardill et al., 2015) and dragonflies (Lin and Leonardo, 2017). In contrast, ambush predators that attack stationary prey in close proximity often use stereopsis, the ability to perceive the world in three dimensions, using the overlapping fields of view of their eyes (Cartmill, 1974; Nityananda and Read, 2017). For example, praying mantids assess the distance to prey using stereopsis (Rossel, 1983; Nityananda et al., 2016). Because stereopsis relies on the interocular visual disparity, the greater the distance between the eyes and the higher their spatial resolution, the more accurate the distance assessment is and the longer its effective range (Burkhardt et al., 1973). When a target is stationary but outside an animal's stereopsis range, distance information can still be gathered via motion parallax. Motion parallax refers to the apparent relative movement of stationary objects created by the translation of the observer (Kral, 2003; Nawrot et al., 2014), with nearby objects appearing to move farther and faster than distant objects. For example, before jumping across gaps, locusts (Wallace, 1959; Collett, 1978), mantids (Poteser and Kral, 1995) and crickets (Goulet et al., 1981) perform peering movements to produce motion parallax information and infer the distance to their target.

**Fig. 1.**
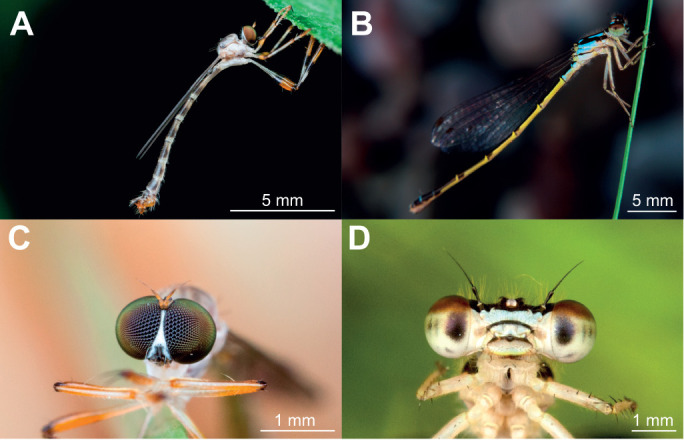
**Pixie robber flies and damselflies are gleaning predators that convergently evolved similar body morphology, but markedly different eyes.** (A) Profile picture of a ringed pixie robber fly (*Psilonyx annulatus*). (B) Profile picture of a fragile forktail damselfly (*Ischnura posita*). (C) Frontal picture of a ringed pixie robber fly. (D) Frontal picture of a fragile forktail damselfly.

Some predators use stereopsis to hunt prey (Rossel, 1983; Yamawaki, 2000, 2003; Nityananda et al., 2016), while others combine both stereopsis and motion parallax (Fox et al., 1977; van der Willigen et al., 1998, 2002; Ohayon et al., 2006; Fux and Eilam, 2009; O'Rourke et al., 2010). As motion parallax requires the observer to move, it has been assumed that relying on it alone might be disadvantageous for predators, as it could alert prey to their presence. Consequently, we are not aware of any predator that has been shown to exclusively use motion parallax when hunting. However, gleaning predation might provide ideal conditions for predators to discreetly use motion parallax to assess prey. During a gleaning attack, static prey resting on a substrate is plucked away by a flying predator. As the gleaning predator is already airborne, it could feasibly gather distance cues via both motion parallax and stereopsis. Some damselflies, suborder Zygoptera (Corbet, 1999; von Reyn et al., 2014; Chai et al., 2025), and pixie robber flies, subfamily Leptogastrinae (Martin, 1968; Newkirk, 1963; Dennis and Lavigne, 1976), are gleaners. These predators provide an excellent comparative model to address the use of motion parallax versus stereopsis while airborne; their body and wing morphologies are strikingly similar (Fig. 1A,B), but their external eye morphologies are not (Fig. 1C,D).

As is typical for predators that use stereopsis (Cartmill, 1974; Nityananda and Read, 2017), damselflies have large interocular distances for their head size (Schröder et al., 2018; Supple et al., 2020) and distinct acute zones in their eyes (Scales and Butler, 2016). The stereopsis range for damselflies has been calculated to be 175 mm, a limit below which they have been found to initiate the majority of their attacks (Schröder et al., 2018; Chai et al., 2025). Damselflies also possess binocular descending neurons with a receptive field matching their preferred prey angle when attacking (Supple et al., 2020). Optical, neuronal and behavioural evidence hence strongly suggests that damselflies rely on stereoscopic information when gleaning. In contrast, pixie robber flies have eyes very close to the midline of their small head, a characteristic which departs from the morphology of the majority of robber fly species (e.g. Lee et al., 2011; Wardill et al., 2017; Talley et al., 2023). Contrary to damselflies, the optical ability of pixie robber flies has not been studied before, leaving open the question of how they can capture similar prey to damselflies with such a small interocular distance.

We hypothesised that, while damselflies use binocular vision, pixie robber flies apply motion parallax to estimate the distance and/or size of their prey. We reasoned that if this was the case, the differences would be reflected in their flight trajectories leading to the attack. To investigate this, we recorded the predatory gleaning behaviour of the ringed pixie robber fly *Psilonyx annulatus* and the fragile forktail damselfly *Ischnura posita*, when presented with natural and artificial prey. The results showed that when assessing prey, pixie robber flies initiated attacks outside their anatomical stereopsis range and showed lateral movements that are absent in the damselfly attack sequence. However, our findings also indicate that pixie robber flies use absolute prey size, not the angular size subtended on the retina, to determine the distance at which to attack prey. Together, these findings suggest that pixie robber flies use motion parallax to assess prey distance or size before attacking. Behavioural patterns during prey assessment, such as moving more and for longer when assessing larger prey, are consistent with the use of motion parallax in other insects (Collett, 1978; Poteser and Kral, 1995; Kral, 1998, 2003). Because such movements are absent in the predatory flights of damselflies, damselflies probably rely on binocular information instead, as the distance from their prey throughout their attack in this and other studies is within their stereopsis range (Schröder et al., 2018; Chai et al., 2025) and they possess visual descending neurons responsive to binocular input (Supple et al., 2020).

## MATERIALS AND METHODS

### Animals

Ringed pixie robber flies, *Psilonyx annulatus* (Say, 1823), were collected as adults from the wild in regional parks of York, PA (USA). Collected animals were kept in an indoor tent at 40–50% humidity and 20–25°C temperature, and fed live fruit flies (*Drosophila melanogaster*) in a laboratory at York College of Pennsylvania (USA). Both males and females were used for experiments.

Fragile forktail damselflies, *Ischnura posita* (Hagen, 1861), were acquired as nymphs from an animal supplier (Carolina Biological Supply Company, Burlington, NC, USA). Nymphs were kept in pond water and fed live blackworms (*Lumbriculus variegatus*) until their final moult. Adult damselflies were kept in an indoor tent at 65–70% humidity and 20–30°C temperature, and fed live fruit flies. Both males and females were used for experiments.

### Indoor testing

Pixie robber flies were collectively transferred to a custom-made transparent 300×195×205 mm^3^ (height×width×depth) tank made of 3 mm thick acrylic boards (Perspex^®^, Mitsubishi Chemical Lucite Group Ltd, Tokyo, Japan), placed vertically on a table. The arena was illuminated with fluorescent tube lights directly above and was placed in front of a white board, illuminated with infrared light, to increase the contrast of the flies in the camera without affecting ambient light. In these conditions, pixie robber flies were recorded responding to two types of stimuli. To test responses to natural prey, live fruit flies of different species and sizes (*Drosophila melanogaster*, *Drosophila affinis* or *Drosophila virilis*) were released into the arena. To test responses to artificial prey, we placed a computer monitor outside the transparent wall of the arena and used it to display stationary targets. The monitor (Apple Inc., Cupertino, CA, USA) measured 180×288 mm^2^ (1600×2560 pixels) with a refresh rate of 60 Hz. Stimuli were created using the GNU Image Manipulation Program (version 2.8.22, The GIMP Development Team) and included colour or greyscale images of ovals and *D. melanogaster* photographs scaled to five sizes (1.58, 2.81, 3.09, 4.33 and 6.53 mm in length). These sizes spanned substantially smaller to larger targets than natural prey: when converted to surface area, they corresponded to approximately 0.5×, 1.3×, 1.5×, 3× and 7× the surface area of a real *D. melanogaster*. These values included a denser sampling around the expected natural prey size to better resolve behavioural responses in this range. Multiple objects of the same type and size were presented simultaneously to maximise the chance of filming an attack.

Damselflies were collectively transferred to either the 300×195×205 mm^3^ tank described above, or a 1×1×1 m^3^ arena made of 3 mm thick acrylic boards (Perspex^®^, Mitsubishi Chemical Lucite Group Ltd), to capture behaviour both in the same spatial constraints and scaled to approximately body size. The arena was illuminated with fluorescent tube lights directly above. The damselflies hunted live fruit flies (*D. melanogaster*) present in the arena.

### Videography and digitisation

We recorded videos at a rate of 1000 frames s^−1^ using two time-synchronised SA2 or WX100 Photron cameras (Photron Ltd, Tokyo, Japan). The system was calibrated using an altered version (Wardill et al., 2017) of J. Y. Boguet's Laboratory's MATLAB toolbox (Caltech, https://doi.org/10.22002/D1.20164), running on MATLAB R2014a (version 8.3, MathWorks Inc., Natick, MA, USA).

The movements of the thorax and abdomen of pixie robber flies and damselflies, as well as the body position of their target, were digitised offline using supervised automatic tracking software (Wardill et al., 2017). The digitised coordinates for each camera were then analysed by a stereo software to reconstruct the three-dimensional trajectory of the object of interest. Both the tracking and the reconstruction were run with MATLAB 2012a (version 7.14).

The three-dimensional coordinates were then smoothed using a Savitzky–Golay filter (Savitzky and Golay, 1964) (polynomial order=3, window size between 101 and 251, manually selected according to trajectory length and noise); the third dimension, or depth, was smoothed twice with the same filter, because of the additional noise due to the stereo calculations. The filter was applied using MATLAB R2018b (version 9.5) and was preferred to other smoothing algorithms (e.g. Dey and Krishnaprasad, 2012) because of its greater speed.

### Kinematic analysis

**Fig. 2.**
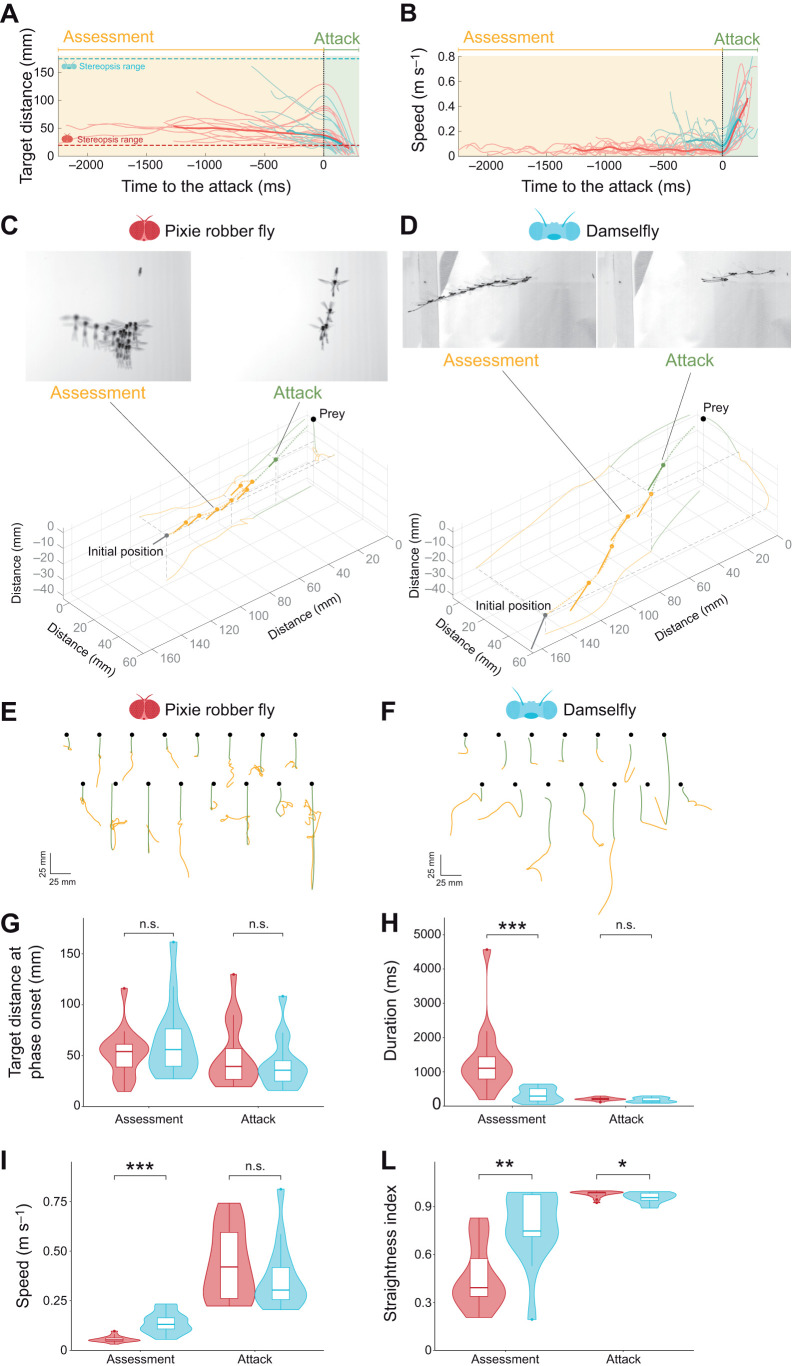
**Variability and phase division in the hunting behaviour of pixie robber flies and damselflies attacking static fruit flies.** (A) The distance from prey over time for pixie robber fly attacks (thin red lines) and damselfly attacks (thin blue lines). The stereopsis limit of pixie robber flies (dashed red line) and of damselflies (dashed blue line) is indicated. (B) Speed plots over time for pixie robber fly attacks (thin red lines) and damselfly attacks (thin blue lines). Thick lines in A and B show the mean profiles for each predator, smoothed. The attack phase (green) was identified as the final acceleration towards the static target. The assessment phase (yellow) preceded the attack phase. (C) Top: two 50 ms overlays of a pixie robber fly attack towards a static fruit fly, one for frames within the assessment phase and one for frames within the attack phase*.* Bottom: a digitised trajectory (dotted line) of a pixie robber fly hunting a static fruit fly (black dot). The position of the fly every 100 ms is shown as a point (thorax) and line (abdomen, actual length), colour coded according to its behavioural phase classification, with assessment in yellow and attack in green. The trajectory is projected on each of the three two-dimensional planes (solid orange/green lines). (D) As in C but for damselflies. (E) Top view of pixie robber flies attacking static fruit flies (black dots). Pixie robber flies' trajectories are colour coded to indicate the behavioural phase (assessment in yellow and attack in green). (F) As in E, but for damselflies. (G) The target distance at the initiation of the assessment and attack phase for each predator. (H) Duration of the assessment and attack phase for each predator. (I) Average speed during the assessment phase and peak speed during the attack phase, measured for each predator. (L) Straightness index of the assessment and attack phase for each predator. Violin plots in G–L show median, upper and lower quartiles and 1.5× interquartile range. Significance was assessed by Wilcoxon rank sum tests: n.s. *P*>0.05, *P<0.05, **P<0.01, ***P<0.001. For all panels, *n*=16 attacks for pixie robber flies, *n*=14 attacks for damselflies.

All kinematic analysis was run offline on smoothed trajectories, using MATLAB 2019b (version 9.7). The flights were trimmed so that they started when the angle formed between the predator's body (abdomen tip to head) and a vector from the abdomen tip to the target was less than 20 deg (Fig. S1), as we found this to be a reliable indicator that the predator had detected the target and was facing towards it. Flights where the body angle started below 20 deg were discarded from the analysis. The shortest distance between the target and the predator's head was taken as the end of the attack (Fig. 2A). The position of the target was taken as the median value for each axis to minimise the impact of errors in the digitisation.

We calculated the fly's speed as the derivative over time of its position, and then smoothed this vector with a Savitzky–Golay filter (polynomial order=3, window size=101). The fly's acceleration was taken as the derivative over time of the speed, smoothed again with the same Savitzky–Golay filter parameters. We found the minima in the speed and considered the last minimum of the flight as *t*=0, the time point determining the end of the assessment phase and the beginning of the attack phase (Fig. 2B).

The straightness index of each phase was calculated as the ratio between the distance from the beginning to the end of the phase and the path length travelled between the two time points (Benhamou, 2004). The mean value of the predatory fly's coordinates along each spatial axis during the assessment was taken as the central point of the flight volume covered by the predatory fly; throughout this paper, we refer to this point as the centroid of the assessment. At each time point, we calculated the distance between the predator and the centroid and used the standard deviation of this distance over the whole trajectory as an estimate of flight movement range. When calculating the perceived size of a target by a predator during a predatory sequence, we used its angular size (δ), defined as δ=2arctan(*d*/2*s*), where *d* is the major diameter (length) of the target and *s* is the distance between the target and the predator.

Pixie robber flies were also recorded spontaneously hovering for short sequences in the arena. To compare flight capability in prey assessments with these hovering sequences despite their different durations, the assessment flight sequences were trimmed to match the mean duration of hovering flights, unless their duration was shorter. Where possible, the time window was trimmed so that the time point of minimal flight speed would be at the midpoint of the trimmed section. When the time point of minimal flight speed was too close to the end of the assessment, a window of the same length was taken from the end of the assessment.

### Microscopy imaging

Protocols for pixie robber fly head fixation and imaging were adapted from previous imaging work on another robber fly (Wardill et al., 2017). In summary, we dissected and fixed whole heads for 48 h in a 4% paraformaldehyde (PFA) and phosphate-buffered saline (PBS) solution, at room temperature. After three PBS rinses, the heads were bleached for 7 days in 35% hydrogen peroxide to clear the cuticle and eyes from screening pigments (Smolla et al., 2014), then rinsed again in PBS. They were then placed in ascending solutions of PBS-diluted ethanol (30%, 50%, 70%, 80%, 90%, 95% and absolute ethanol) and subsequently the same solutions in reverse. Finally, they were transferred through an ascending dilution series of 2,2′-thiodiethanol (TDE) and PBS (from 10% to 90% TDE in steps of 10, and a final concentration of 97% TDE solution) (Gonzalez-Bellido and Wardill, 2012). We then imaged the whole heads with an oil-immersion objective (Olympus XLSLPLN25XGMP, Tokyo, Japan), using 97% TDE solution as mounting medium. A Bruker two-photon microscope (Billerica, MA, USA) was used to image tissue autofluorescence to 810 nm light, produced by a Spectra-Physics Insight^®^ DS+ TM laser (Santa Clara, CA, USA). The image voxel resolution was 0.4 µm^3^.

Eye cross-sections for transmission electron microscope (TEM) imaging were obtained by preparing specimens as done in previous literature (Meinertzhagen and O'Neil, 1991; Meinertzhagen, 1996; Gonzalez-Bellido et al., 2011). Heads were fixed in a modified Karnovsky fixative, containing 2.5% glutaraldehyde and 2.5% PFA aqueous solution diluted in cacodylate buffer (Shaw et al., 1989), before being rinsed in PBS three times. Samples were left in 2% osmium tetroxide and 0.2 mol l^−1^ cacodylate buffer solution for 2 h and dehydrated in a 30 min step alcohol series (50%, 70%, 80%, 90%, 95% and two absolute ethanol immersions), followed by a 30 min immersion in absolute acetonitrile. We then placed the heads in a 50% mix of acetonitrile and Epon resin (Poly/Bed 812, PolySciences, Warrington, PA, USA) and left them, with agitation, to allow the acetonitrile to evaporate overnight. Following this, we placed the heads in Epon resin with agitation overnight. Finally, we embedded the heads in fresh resin left at 60°C overnight. We sectioned the embedded heads into 70 nm thin cross-sections centred on the 13 largest facets of one of the two eyes, which we considered as that eye's acute zone, or fovea. The sections were then transferred over copper disks, coated with 2% Formvar (Sigma-Aldrich, St Louis, MO, USA), and stained with 3% uranyl acetate and Renolds lead citrate. The grids were imaged using a JEOL JEM-1400Plus 120 kV TEM.

### Visual parameter estimation

Visual acuity parameter estimation of the pixie robber flies was based on previous literature on fruit flies and robber flies (Stavenga, 2003; Wardill et al., 2017). Using ImageJ (National Institutes of Health, Bethesda, MD, USA), we rotated the two-photon scans on all axes along the head's midline, so that the robber fly's largest two lenses had their inner surface parallel to the head's cross-section. For each lens in the acute zone, we calculated the fly's facet lens diameter (*D*_f_) by averaging the three diagonals of the hexagonal crystalline cone under each lens (Wardill et al., 2017). We then resliced the scan to have a coronal view of the retina. For each lens, we used a coronal section through the lens's centre to calculate the optical features of the lens (Stavenga, 2003). From these sections, it appeared that the inner (image space) surface of the facet lenses is approximately flat, resulting in distinctive optics (see Supplementary Materials and Methods).

For optimal performance, the rhabdomere tips of the photoreceptors have to be in the image focal plane of their ommatidium's facet lens. To assess whether that was realised, we estimated the distance of the rhabdomere tips to the inner surface of their facet lens, which is given by:
(1)

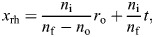
where *n*_o_, *n*_f_ and *n*_i_ are the refractive indices of object space, facet lens and image space, respectively, *r*_o_ is the curvature of the outer (object space) lens surface and *t* is the lens thickness (Fig. S2).

As the focal distance in object space is:
(2)

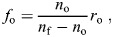
the lens' *F*-number is obtained with the facet lens diameter *D*_f_:
(3)

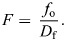


In the neural superposition eyes of flies, the interommatidial angle Δϕ ideally equals the angle between the optical axes of neighbouring visual sense cells within an ommatidium, which is given by *s*_rh_/*f*_o_, where *s*_rh_ is the mutual separation of neighbouring rhabdomere tips, which are located in the image space focal plane (see Supplementary Materials and Methods). However, a more realistic estimate is Δϕ=0.8*s*_rh_/*f*_o_ (Pick, 1977).

Using the acquired transmission electron micrographs of the photoreceptor tip (Wijngaard and Stavenga, 1975), we fitted ellipses around each rhabdomere of the ommatidia in the fovea. We averaged the major and minor axes of the ellipse and used this as a measure of photoreceptor diameter (*D*_rh_). The acceptance angle of the photoreceptor cells depends on many factors, for instance the facet lens' focal length, the rhabdomere diameter, the light wavelength and the visual pigment's absorption spectrum (Stavenga, 2003), but a simple first measure is the angle subtended by the rhabdomere Δρ=*D*_rh_/*f*_o_ (Stavenga, 2003).

Finally, we measured the distance between the biggest lenses on the right and left eye (*s*_fovea_) to calculate the flies' stereopsis range (*E*_∞_) (Burkhardt et al., 1973):
(4)

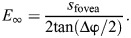


### Data analysis and statistics

Statistical tests were run using RStudio software, version 1.2.5001 (https://posit.co/), and R software, version 3.6.1 (http://www.R-project.org/). In this study, the mean and s.e.m. are used as descriptive statistics. However, when the data were non-normally distributed, the median and interquartile range (IQR) are given instead. The interquartile range was calculated using the *basic* package. Means, medians and standard deviations were calculated using the *pastecs* package, as was data normality testing using the Shapiro–Wilk test. Variance homogeneity was tested using Levene's test, within the *car* package. Significance value (*P*) was 0.05 for all tests, except where *P*-values were adjusted.

To test whether a value was part of a non-normal data distribution, we used a one-sample Wilcoxon signed rank test in the *stats* package. For comparisons between two groups with normally distributed data and homogeneous variance between the groups, we used a two-sample Student's *t*-test, also in the *stats* package. If the data were non-normally distributed or if the variance was heterogeneous, we used a Wilcoxon rank sum test in the *stats* package. For comparisons between multiple groups with normally distributed data and homogeneous variance amongst the groups, we used the Analysis of Variance (ANOVA) in the *stats* package. If data were not normally distributed or the variance was heterogeneous, we used a Kruskal–Wallis test in the *stats* package. Linear models (LMs) in the *stats* package were used to implement linear regressions to data with normal error distribution and homogeneous variance. If data had non-normal error distributions or heterogeneous variances, generalised linear models (GLMs), also in the *stats* package, were implemented instead.

## RESULTS

### The attack strategies of damselflies and pixie robber flies show marked differences when assessing prey

Both ringed pixie robber flies, *P. annulatus*, and fragile forktail damselflies, *I. posita*, hunted static fruit flies in our arena by orienting their bodies towards the prey once it was detected (Movie 1). Because both species are airborne before they detect prey, the start of the behavioural sequence was marked when the angle between the predator's body and the prey first fell below 20 deg, a reliable sign that the potential prey had been detected (Fig. S1). Both robber flies (*n*=16) and damselflies (*n*=14) showed a consistent pattern of flight speed and distance from the target across their trajectories, which divided the predatory sequence in two phases (Fig. 2A–D). In the first phase, which we call the assessment phase, the predators maintained a low speed, with their body oriented towards their target. In the second phase, which we call the attack phase, they rapidly accelerated towards the target, capturing it with their legs and pulling it away from the substrate for consumption (Movie 1).

Although the overall structure of the predatory behavioural sequence was similar between the two species, their flight trajectory varied substantially (Fig. 2E,F, Table 1; Fig. S3). The distance of the two predators from their targets at the beginning of the hunting sequence was not significantly different (Fig. 2A,G), indicating that the two species detected prey at similar distances. However, when compared with that of damselflies, the robber flies' assessment phase had a longer duration (Fig. 2H) and lower speed (Fig. 2B,I). In addition, the flight path during the assessment phase appeared more convoluted in pixie robber flies than in damselflies (Fig. 2E,F). To quantify this, we calculated the straightness index for both predators; a straightness value of 1 means completely straight; the closer to 0, the more tortuous the trajectory. The robber flies' assessment paths were significantly more tortuous than those of damselflies (Fig. 2L). In addition, when presented with unsuitable targets such as conspecifics and flat pieces of paper, pixie robber flies assessed the potential prey, but withheld the attack (example shown in Fig. S4, Movie 2). This suggests that the pixie robber flies' tortuous assessment may play an active role in evaluating the suitability of the potential targets.

**Table 1.** Statistical comparison of the hunting behaviour of pixie robber flies and damselflies

	*Psilonyx annulatus*		*Ischnura posita*		*W*	*P*
	Value	Variance	Value	Variance		
Assessment phase						
Target distance at onset (mm)	53.9	22.3	55.9	36.7	130	0.467
Duration (ms)	1105	647	292	360	15	<0.001
Speed (m s^−1^)	0.06	0.01	0.14	0.02	215	<0.001
Straightness index	0.39	0.24	0.75	0.26	185	0.003
Attack phase						
Target distance at onset (mm)	39.3	30.2	35.6	20.1	97	0.547
Duration (ms)	207	11	176	19	79	0.177
Peak speed (m s^−1^)	0.42	0.33	0.30	0.16	86	0.289
Peak acceleration (m s^−2^)	3.97	1.49	3.53	3.04	103	0.724
Straightness index	0.99	0.02	0.96	0.05	59	0.029

Values and variance are shown as means and s.e.m. (assessment phase speed and attack phase duration) or as medians and interquartile range (all other parameters). Sample size: *n*=16 attacks for pixie robber flies (*P. annulatus*), *n*=14 attacks for damselflies (*I. posita*). Data were assessed by Wilcoxon rank sum test (*W*) and significance (*P*-value) is shown.

In contrast to the assessment phase, the attack phase was very similar between robber flies and damselflies (Table 1). There were no significant differences for (i) the distance from the prey at which the predator initiated the attack (Fig. 2G), (ii) the duration of the attack (Fig. 2H), (iii) the peak speed (Fig. 2I) and (iv) the peak acceleration (Table 1). The straightness of the attack trajectory was close to 1 (straight line) for both species, with robber flies having straighter attacks than damselflies. This was the only significantly different measure of the attack between the two predators (Fig. 2L).

Altogether, the results above indicate that pixie robber flies and damselflies have key differences in their prey assessment strategies. Robber flies assessed their prey by performing higher-tortuosity, lower-speed manoeuvres than damselflies. In contrast, the attack phases of the two species were similar in duration, speed and acceleration, both drawing almost straight paths towards their prey.

### Pixie robber flies span a smaller movement range when hovering than during prey assessment

The tortuosity displayed by the pixie robber flies during the prey assessment phase is three-dimensional (Fig. 2C), so that the body of the animal appears to translate around an imaginary centre point, without changing its body orientation (Fig. S1). Pixie robber flies might fly in such a way during prey assessment because they are incapable of a more stable flight. To quantify their flight performance, we recorded pixie robber flies while they spontaneously hovered in the arena when not engaged in predation (*n*=10 flights; Fig. S5) (Newkirk, 1963), and compared their body displacement during hovering with that during prey assessment (*n*=16 flights). The minimal flight speed of hovering flights detected with our setup was significantly lower than the minimal flight speed reached during assessment flights (hover: 3.9±0.8 mm s^−1^; assessment: 11.9±1.8 mm s^−1^, means±s.e.m.; Wilcoxon rank sum test, *W*=18, *P*=0.001), indicating that flight instability was not the cause of the body translations seen during assessment flights. However, hovering sequences were of shorter duration than assessment flights [hover: 373 ms (165 ms); assessment: 1292 ms (717 ms), median (IQR); Wilcoxon rank sum test, *W*=18, *P*=0.001]. To control for this difference, the assessment flight sequences were trimmed as outlined in the Materials and Methods, to match the mean duration of hovering flights (392 ms), unless their duration was shorter. We then calculated the movement range as the standard deviation of the fly's distance from the centre of the flight volume of this trimmed portion. In accordance with the results from full assessment flights, these trimmed flight sections had a significantly higher movement range [0.896 mm (0.77 mm), median (IQR)] than hovering flights [0.523 mm (0.42 mm); Wilcoxon rank sum test, *W*=35, *P*=0.019]. As the body displacement during hovering flights is much lower than during prey assessment flights, we infer that the tortuous flight path in the assessment phase is unlikely to arise from a lack of flight control. Instead, we suggest that the body displacements performed during the assessment phase serve an active role in prey assessment. Next, we investigated what cues might be obtained during such assessment phases.

### Two-dimensional targets trigger pixie robber fly attacks

During the tortuous flightpath of their assessment phase, pixie robber flies could acquire relief information such as texture, shading or shadows cast by the three-dimensional body of their prey, which could make camouflaged prey emerge from the background (Gårding et al., 1995; Nityananda and Read, 2017; Galloway et al., 2020; El Nagar et al., 2021). To test this possibility, we presented pixie robber flies with a computer screen that displayed 2.8 mm long photographs of fruit flies on a white background, comparable to the size of real fruit flies (2.45±0.06 mm, mean±s.e.m., *n*=10 animals). The attacks on images (*n*=8 flights) and on real fruit flies (*n*=16 flights) did not differ significantly in any of the parameters measured (distance from prey at the beginning of the assessment, duration and straightness index of the assessment phase, distance from prey at the beginning of the attack, peak speed; Fig. S6). Because of this, relief information on its own cannot be the determinant cue for the outcome of the prey assessment for pixie robber flies.

It may, however, be possible that, whilst incapable of perceiving relief details of their prey, pixie robber flies use assessment flights to detect the difference in the image speed between real prey and its background, called velocity contrast (Collett and Paterson, 1991). This velocity contrast arises as a result of distance-dependent differences in translational optic flow and contains information about the relative distance between the prey and the background. Invertebrates known to use this strategy do so by looking at velocity contrast at the target edge (Osorio et al., 1990; Srinivasan et al., 1990; Collett and Paterson, 1991). As uniform backgrounds do not appear to move, any target image overlayed on a uniform background, as tested above, still produces velocity contrast information. To remove any velocity contrast, we overlaid images of fruit flies on two-dimensional textured backgrounds on a computer screen, i.e. within the same image plane and thus with a velocity contrast of zero. We tested two backgrounds, one picturing foliage and one with netting. Pixie robber flies attacked images of fruit flies on both background pictures (*n*=3 and *n*=9 flights, respectively; Movie 3). The attacks on fruit fly images on the blank and textured background differed significantly only in the target's distance at attack onset (*P*=0.002), with robber flies hunting on textured backgrounds positioning themselves at a longer distance from their prey. Other parameters measured (distance from the prey at the beginning of the assessment, duration and straightness index of the assessment phase, and peak speed during the attack; Fig. S6) did not differ significantly between the two stimuli. This suggests that, while pixie robber flies do not require velocity contrast between a target and its substrate to attack, the absence of velocity contrast affected the distance they kept from their target when launching the attack.

We also tested robber flies for prey shape or colouration preference (Movie 3). Pixie robber flies attacked coloured pictures of fruit flies (*n*=8 flights), black and white pictures of the same size (*n*=3 flights) and grey ovals of the same length and width (*n*=5 flights). The attacks on fruit fly photographs in colour and in greyscale did not differ significantly in any of the parameters measured (distance from the prey at the beginning of the assessment, duration and straightness index of the assessment phase, distance from prey at the beginning of the attack, peak speed; Fig. S6), suggesting that pixie robber flies do not strictly predate on prey of a certain colour or more specific features than a general body outline.

### Effect of prey size on the assessment and attack phases of pixie robber flies

**Fig. 3.**
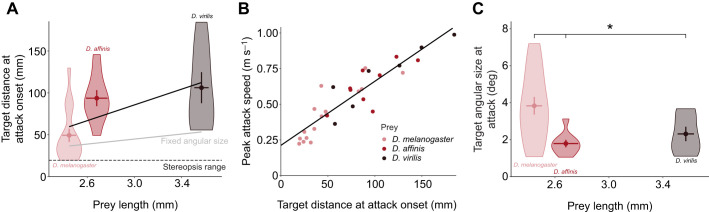
**Attacks of pixie robber flies on real fruit flies of different sizes.** (A) Pixie robber fly distance from each prey species when the attack is launched. The grey line indicates the theoretical distance robber flies would need for prey to subtend the same angular size as the average angular size of *Drosophila melanogaster*. The dashed line represents the stereopsis range of the pixie robber fly (see Materials and Methods). (B) Pixie robber fly peak speed during attack on three prey species as a function of the distance between the robber fly and its prey when the attack is launched. (C) Angular size of each of the three prey species when the pixie robber fly launches the attack. In A and C, dots and bars represent means and s.e.m. for each axis. For all panels, *n*=16 attacks for *D. melanogaster*, *n*=9 attacks for *D. affinis*, *n*=7 attacks for *D. virilis*. Solid black lines represent significant generalised linear models (GLMs). Significance in C was assessed by a Kruskal–Wallis test: *P<0.05.

As prey were attacked even in the absence of velocity contrast, it is possible that the motion parallax information obtained during tortuous assessment flights allows pixie robber flies to estimate prey distance and/or size. Hence, we tested whether prey size was a key cue for the attack. In the wild, pixie robber flies hunt a variety of prey, ranging from flies to spiders (Newkirk, 1963). In our experimental arena, this robber fly species readily attacked real fruit flies of different sizes (*d*): *D. melanogaster* with body length *d*_Dm_=2.45±0.05 mm (mean±s.e.m., *n*=10 animals), *Drosophila affinis* with *d*_Da_=2.68±0.09 mm (*n*=10 animals) and *Drosophila virilis* with *d*_Dv_=3.57±0.06 mm (*n*=10 animals). Notably, the attacks to capture *D. melanogaster* (*n*=16 flights), *D. affinis* (*n*=9 flights) and *D. virilis* (*n*=7 flights) were launched at a progressively greater distance from the target and this was correlated with prey size (GLM, *t*=3.11, *P*=0.004; Fig. 3A). An attack that is initiated at a greater distance from the prey allows additional time for acceleration. Accordingly, peak speed was significantly higher for larger prey (LM, *t*=2.83, *P*=0.008) and peak speed during the attack was dependent on the distance from the target when the attack was launched (GLM, *t*=11.5, *P*<0.001; Fig. 3B). The distance at which pixie robber flies position themselves before launching an attack could be explained by a simple heuristic, such as approaching until the target reaches a fixed subtended angle on the retina. In this case, a larger target would reach this size threshold at a greater distance than would a smaller target. However, when the attack was launched, the subtended angular sizes (δ) of the three *Drosophila* species were significantly different (*D. melanogaster*: δ_Dm_=3.83±0.48 deg; *D. affinis*: δ_Da_=1.78±0.20 deg; *D. virilis*: δ_Dv_=2.31±0.39 deg, means±s.e.m.; Kruskal–Wallis test; χ^2^_2_=8.33, *P*=0.016; Fig. 3C).

**Fig. 4.**
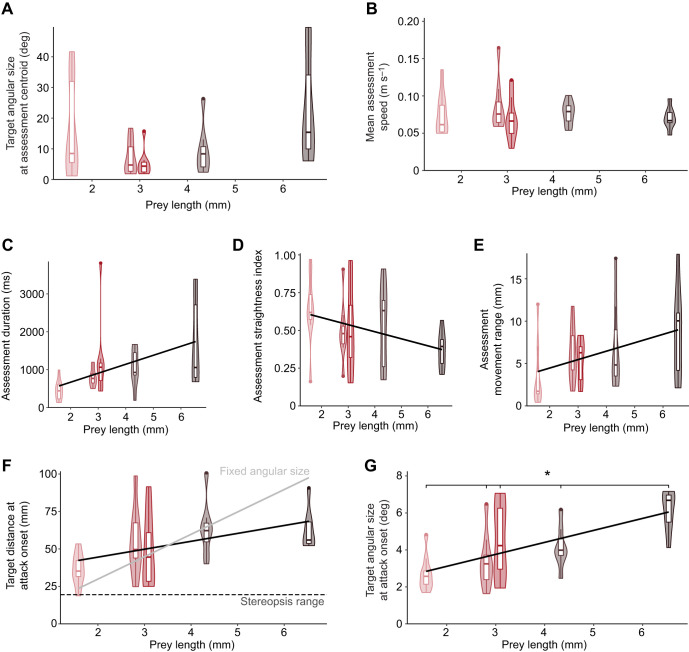
**Predatory sequences of pixie robber flies with virtual target images of different sizes.** (A) Angular size of targets of different sizes at the centroid of pixie robber fly assessment. (B) Mean flight speed of pixie robber flies while assessing prey of different sizes. (C) Duration of the pixie robber fly assessment for prey of different sizes. (D) Straightness index of pixie robber fly assessment for prey of different sizes. (E) Standard deviation of distance from the central point of pixie robber fly assessment (i.e. the movement range) for prey of different sizes. (F) Pixie robber fly distance from prey of different sizes when the attack is launched. The grey line indicates the theoretical distance robber flies would need for prey to subtend the same angular size as the average angular size *D. melanogaster* subtends in Fig. 3A; the dashed line represents the stereopsis range of the pixie robber fly (see Materials and Methods). (G) Angular size of prey of different sizes when pixie robber flies launch the attack. Violin plots show median, upper and lower quartiles and 1.5× interquartile range. For all panels, *n*=9 attacks for prey size 1.58 mm, *n*=8 attacks for prey size 2.81 mm, *n*=8 attacks for prey size 3.09 mm, *n*=7 attacks for prey size 4.33 mm, *n*=7 attacks for prey size 6.53 mm. Solid black lines represent significant GLMs. Significance in G was assessed by a Kruskal–Wallis test: *P<0.05.

To further investigate the role of prey size while controlling for other variables such as colour and contrast, we recorded pixie robber flies hunting the same image of a *D. melanogaster* presented on a screen and scaled such that its body length was 1.58 mm (*n*=9 flights), 2.81 mm (*n*=8 flights), 3.09 mm (*n*=8 flights), 4.33 mm (*n*=7 flights) or 6.53 mm (*n*=7 flights). For each predatory flight, we found the centroid of the assessment phase as outlined in the Materials and Methods and calculated the target's angular size from this point. At the centroid point, the angular size of the target did not correlate with the target's absolute size [target's angular size at central assessment point: 6.69 deg (10.0 deg), median (IQR), *n*=39; GLM, *t*=1.50, *P*=0.144; Fig. 4A]. The robber flies’ mean speed during the assessment did not differ between attacks on prey of different sizes (ANOVA, *F*_4,34_=0.569, *P*=0.687; Fig. 4B). However, larger targets elicited longer and more tortuous assessment flights (GLM, *t*=3.28, *P*=0.002; and LM, *t*=2.10, *P*=0.042; Fig. 4C,D, respectively). The movement range, i.e. standard deviation from the centroid of the assessment, was also higher in response to larger prey (LM, *t*=2.55, *P*=0.015; Fig. 4E) and was correlated with the distance between the target and the centroid of the assessment (GLM, *t*=3.89, *P*<0.001).

After the assessment phase and just prior to attack onset, the distance at which pixie robber flies positioned themselves from their prey was correlated with the prey's absolute size (LM, *t*=2.85, *P*=0.007; Fig. 4F). Crucially, the targets subtended different angular sizes at the position where the attack was launched (Kruskal–Wallis test; χ^2^_4_=16.13, *P*=0.003; Fig. 4G) and these angular sizes were correlated with absolute prey size (GLM, *t*=4.71, *P*<0.001). This suggests that, by the time the assessment was completed, the target's absolute size, not its angular size, was used to determine at what distance to launch the attack.

To summarise, larger prey were assessed by robber flies at a greater distance than smaller prey, so that they would subtend a similar angular size. Larger prey elicited longer and more tortuous assessment flights, which were performed with a larger movement range but similar flight speeds. Following an assessment, the position chosen by the robber fly to initiate the attack phase was correlated with the target's absolute size, not with its angular size. As the target's angular size at the beginning of the attack was not the same for larger and smaller prey, a simple heuristic rule was not used to determine what distance to attack from.

### Prey is outside the pixie robber fly's stereopsis range when an attack is launched

**Fig. 5.**
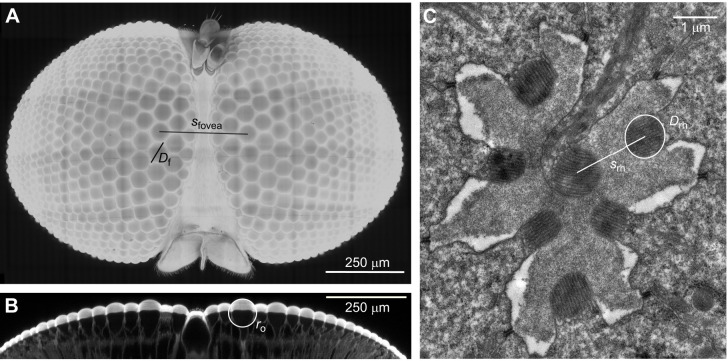
**Optical measurements from pixie robber fly eyes.** (A) Two-photon scan of a pixie robber fly head showing the enlarged frontal facets close to the midline. The black lines show the measurement of facet lens diameter (*D*_f_) and interfoveal distance between the two eyes (*s*_fovea_). (B) Coronal section of the two-photon stack showing a circle fitted to the distal (object) curvature of a lens, the radius (*r*_o_) of which was used as a measurement. (C) Transmission electron micrograph showing the cross-section of an ommatidium in the acute zone. The ellipse fitted to one of the photoreceptors is shown, as well as the distance between the centroids of rhabdomeres R7 and R1 (*s*_rh_).

To test whether pixie robber flies could use stereopsis during prey assessment, we investigated the capability of their visual system (Fig. 5A). Using two-photon microscopy, we imaged the 13 biggest facet lenses of each eye (*n*=3 animals, *n*=78 lenses). From the resulting three-dimensional stack, a coronal view was taken through each lens (Fig. 5B). Lenses were curved on the outer (object) surface and flat on the inner (image) surface. We fitted a circle to the outer surface of each lens and measured its radius. Using this radius (*r*_o_=40.9±0.3 µm, mean±s.e.m.), the refractive index of air (*n*_o_=1.00) and the refractive index of the lens (*n*_f_=1.43; Stavenga et al., 1990), we calculated the power of the lens, *P*_f_=10.5 mm^−1^. We then used these values to calculate the object space focal length, *f*_o_=95.1 µm, and the image space focal length, *f*_i_=127.5 µm, using 1.34 as the refractive index of the image space, *n*_i_ (Seitz, 1968). The object space focal length was divided by the median diameter of the facet lenses (*D*_f_=68.6±0.45 µm) to calculate the lens' *F*-number, *F*=1.39.

To validate these findings, we measured the thickness of each lens (*t*=31.6±0.2 µm) and used this to calculate the position of the focal plane where the rhabdomere tips of each ommatidium would be expected to be located (*x*_rh_=97.7±0.6 µm; see Supplementary Materials and Methods). The distance between the proximal surface of the lens and the tip of the rhabdomeres, measured using two-photon scans (93.9±1.3 µm), was not significantly different from the image focal distance *x*_rh_ as calculated above (Wilcoxon rank sum test, *W*=2573, *P*=0.097). Thus, both calculations are supportive of a focal plane positioned approximately 94–98 µm from the inner surface of the lens.

Transmission electron micrographs of the robber fly's ommatidia were taken at the distal portion of the fovea's rhabdoms (Fig. 5C; Fig. S7). We calculated the average between the two axes of an ellipse fitted to the cross-section of each rhabdomere (*D*_rh_: 0.779±0.023 µm, *n*=5 animals measured for one ommatidium each, *n*=35 rhabdomeres) and divided this by the object space focal length to calculate the geometrical acceptance angle, Δρ=0.47 deg (see Supplementary Materials and Methods). The electron micrographs were also used to extract the distance distally between the rhabdomere axes (*s*_rh_: 1.74±0.16 µm, *n*=5 animals measured for one ommatidium each, *n*=25 rhabdomere distances). We divided this value by the object space focal length, and applied Pick's correction (Pick, 1977), which gives the interommatidial angle of the pixie robber fly's fovea, Δϕ=0.84 deg. We measured the distance between the biggest lenses from the two-photon scan [*s*_fovea_: 285 µm (3 µm), median (IQR)]. We divided this number by twice the tangent of half the interommatidial angle, to approximate the stereopsis range of pixie robber flies, *E*_∞_=19.5 mm (results summarised in Table 2).

**Table 2.** Summary of the morphological measurements taken from the pixie robber fly head and the subsequently calculated features of the fly's visual system

Parameter	Abbreviation	Value
Lens object space radius	*r*_o_	40.9±0.3 μm
Facet diameter	*D*_f_	68.6±0.45 μm
Rhabdomere diameter	*D*_rh_	0.779±0.023 μm
Rhabdomere distance	*s*_rh_	1.74±0.16 μm
Interfoveal distance	*s*_fovea_	285 μm
Facet lens power	*P*_f_	10.5 mm^−1^
Object-space focal length	*f*_o_	95.1 μm
Image-space focal length	*f*_i_	127.5 μm
*F*-number	*F*	1.39
Acceptance angle	Δρ	0.47 deg
Interommatidial angle	Δϕ	0.84 deg
Stereopsis range	*E*_∞_	19.5 mm

Data for *r*_o_, *D*_f_, *D*_rh_ and *s*_rh_ are means±s.e.m. Sample size: *n*=3 animals, *n*=78 lenses.

This stereopsis range is significantly shorter than the distance from which the pixie robber fly launched the attack on *D. melanogaster* (*n*=16 flights, 39.3 mm (30 mm), median (IQR), Wilcoxon signed rank test, *W*=135, *P*<0.001), the distance between the assessment flight's centroid and *D. melanogaster* (*n*=16 flights, 42.5±3.7 mm, Wilcoxon signed rank test, *W*=135, *P*<0.001) and the minimal distance between predator and *D. melanogaster* during the assessment (*n*=16 flights, 31.7±3.1 mm, Wilcoxon signed rank test, *W*=129, *P*<0.001). This suggests that during their predatory flights, pixie robber flies are unlikely to use stereopsis to gather three-dimensional information about their prey, such as its size or distance.

## DISCUSSION

In this paper, we show that the predatory sequence of ringed pixie robber flies and fragile forktail damselflies can be divided into two behavioural phases (Fig. 2). In the first phase, which we call the assessment phase, both robber flies and damselflies orient their body and assess their prey. In the second phase, the attack phase, both predators accelerate towards their prey for capture. During the attack, pixie robber flies show similar flight kinematics to those of damselflies. However, robber flies assess their prey for longer, at lower speed and with more convoluted trajectories than damselflies, which show more streamlined trajectories. When not hunting, pixie robber flies are capable of flying at lower speed and with a smaller movement range than when assessing prey (Fig. S5). Therefore, the more convoluted nature of their assessment flights seems functional rather than a reflection of flight instability. Moreover, the fact that robber flies and damselflies performed so similarly during the attack phase suggests that the difference in flight during prey assessment is not due to the physical constraints the smaller robber flies face during flight.

Being generalist hunters (Newkirk, 1963), pixie robber flies seem unlikely to have a strict prey template. This was confirmed in our experiments, where pixie robber flies hunted prey images of different shapes and colours (Movie 3), and in the literature, with descriptions of pixie robber flies hunting inedible objects of similar size to their prey (Newkirk, 1963; Martin, 1968; Dennis and Lavigne, 1976). Instead, we suggest that their tortuous assessments are used to evaluate depth-related information, such as prey size and/or distance. Pixie robber flies readily hunted both real prey and pictures of prey of a range of sizes in our arena (Fig. 3). Attacks towards real prey of larger size were launched from a greater distance, which allowed the robber flies to reach higher peak speeds. High speeds at the end of the attack are possibly needed to produce a large impact force onto the prey, as seen in other predators (deVries et al., 2012). Pixie robber flies may therefore launch the attack towards bigger targets from further away to produce enough force to snatch the prey. Theoretically, if prey of different absolute size subtended the same angular size on the pixie robber fly's retina when the attack was launched, then the robber fly's distance from the target would be larger for bigger prey. Data from attacks towards real prey did not show a clear trend in this regard, although it is worth noting that the natural colouration of *D. melanogaster* is paler than that of both *D. affinis* and *D. virilis*. To investigate this systematically, artificial prey images were used instead of real prey. Pixie robber flies treated prey images shown on a computer monitor as real prey. Data from prey images suggest that pixie robber flies do not determine where to initiate the attack based on a set angular size, as targets of different lengths subtended different angular sizes at the beginning of the attack. This suggests that the distance at which pixie robber flies launch the attack depends on the target's absolute size.

To investigate how pixie robber flies might determine their prey's size, we used morphological data from the robber fly's eyes. We used the focal lengths of the facet lenses and rhabdomere distances to calculate their interommatidial angles (Wardill et al., 2017). Ideally, we would have liked to confirm these results with more established pseudopupil measurements (Petrowitz et al., 2000), but the dark pigments in the pixie robber fly's eyes made this method impossible. Nevertheless, the interommatidial angle (0.83 deg) found in the acute zone of this small fly is comparable to that of larger predators such as damselflies (0.82 deg; Scales and Butler, 2016) and praying mantids (Horridge and Duelli, 1979). Indeed, pixie robber flies and damselflies have a very similar interommatidial angle, which could explain why these two gleaners were able to initiate their prey assessments from a similar distance. We used the pixie robber fly's interommatidial angle and interfoveal distance (under 0.3 mm) to calculate its stereopsis range (∼20 mm). This was much smaller than the distance at which the pixie robber fly assesses prey, suggesting that these animals do not use stereopsis to determine prey size. Considering the convoluted prey assessment pixie robber flies employ, we suggest that they use motion parallax to determine the size of their prey. In contrast, damselflies have an interfoveal distance almost 10 times larger (2.5–3 mm for damselflies of a similar size to the ones used in our experiments; Schröder et al., 2018; Chai et al., 2025), which gives them a stereopsis range above 175 mm (Schröder et al., 2018). Damselflies were found to take off in response to prey within this range by this and other studies (Schröder et al., 2018; Chai et al., 2025), suggesting that binocular vision is heavily involved in their predatory hunts.

The assessment flights of robber flies do indeed have some features in common with those of other animals that use motion parallax. When trying to jump towards a distant object, both locusts (Collett, 1978) and mantids (Poteser and Kral, 1995) perform larger peering movements than when targeting an object nearby, to increase their accuracy. However, the speed of the peering stays constant, which suggests that they use the target's angular speed, rather than its angular displacement, to determine its distance (Kral, 1998, 2003). We saw the same pattern with pixie robber flies. When assessing larger prey, which was farther away, the pixie robber flies' movement range increased and the straightness index decreased, but their average speed stayed constant. Pixie robber flies also assessed bigger prey for longer, which could be a way to compensate for the reduced accuracy of motion parallax when used for farther away prey (Collett, 1978). The general accuracy of motion parallax relies on precise estimation of self-motion, which pixie robber flies could approximate in a similar way to many other flying insects (Taylor and Krapp, 2007), i.e. via airflow sensors, stretch receptors on the forewings, halteres and antennae, and/or via optic flow measurements from the ocelli and compound eyes. For these reasons, we propose that pixie robber flies use motion parallax to determine the size of their prey.

Other insects that use motion parallax (Srinivasan et al., 1989; Collett and Paterson, 1991; Zeil, 1993; Walcher and Kral, 1994; Ravi et al., 2019) are able to use absolute motion parallax, determining the absolute distance of one object, as well as relative motion parallax, to determine how objects are positioned in relation to each other. From our experiments, is not clear how pixie robber flies use relative parallax cues. Pixie robber flies were presented with images of prey on a blank background or the same images of prey on a textured background. For a moving observer, a blank background does not appear to move, while images overlayed on it do. This difference, called velocity contrast (Collett and Paterson, 1991), is equal to zero if the images are overlayed on a textured background instead, as the images and the background have the same apparent speed. Pixie robber flies readily attacked stimuli with zero velocity contrast, showing that a velocity contrast is not needed to attack prey, but they launched their attacks from greater distances. This could be because prey appeared to be closer to the pixie robber flies when overlayed on a textured background, hence the need to increase the attack distance. However, the opposite could also be true; prey could appear to be further away on a textured background but, in order to subtend the same angular size, also appear bigger. Hence, pixie robber flies could be increasing the distance of their attack to produce higher terminal speed. Indeed, for locusts, the apparent distance of targets increases with decreasing velocity contrast (Collett and Paterson, 1991). Because target size and distance both affect the pixie robber fly’s position when launching an attack, further investigation is needed to disentangle the effects of relative motion parallax on either.

The use of motion parallax to determine distance is very widespread in the animal kingdom, from humans to insects (Collett, 1978; Rogers and Graham, 1979; Ellard et al., 1984; Goulet et al., 1981; Davies and Green, 1988; Srinivasan et al., 1989; Poteser and Kral, 1995; van der Willigen et al., 2002). Locusts (Wallace, 1959; Collett, 1978), mantids (Poteser and Kral, 1995) and crickets (Goulet et al., 1981) use motion parallax for jumping. Bees use motion parallax to estimate flower height (Lehrer et al., 1988), the distance and size of landmarks (Srinivasan et al., 1989; Lehrer and Srinivasan, 1994), and the size of gaps before crossing them (Ravi et al., 2019). Similarly, fruit flies use motion parallax to adjust their flight (Ruiz and Theobald, 2020; Shomar et al., 2025). Although most animals probably use a combination of motion parallax and stereopsis with different contributions to match ecological demands (Cao and Schiller, 2002), only the barn owl has been suggested to use motion parallax in conjunction with stereopsis when hunting (van der Willigen et al., 1998, 2002; Ohayon et al., 2006; Fux and Eilam, 2009). Mantids have been shown to perform head movements when attacking, though the amplitude of these movements was found to be too small for range estimation (Yamawaki, 2000, 2003). Motion parallax is not widely used by predators, possibly because the peering movements necessary to produce this type of information might give away their presence, allowing prey to escape.

Pixie robber flies have evolved motion parallax to capture prey, but why have they done so? Their large lenses allow them to spot prey at similar distances to damselflies, but their much smaller heads mean that their stereopsis range is very limited. If pixie robber flies used binocular cues to determine absolute prey parameters, they would be too close, with the risk of being spotted and their prey escaping. Motion parallax provides depth information at a much longer range than stereopsis for pixie robber flies, which could explain why they initiate attacks at absolute distances similar to those observed in damselflies and reach similar peak speeds during the attack. Indeed, motion parallax can be effective over longer distances than stereopsis (Poteser and Kral, 1995), it has a similar sensitivity threshold (Rogers and Graham, 1982), and it could be as effective against prey camouflage (Adams et al., 2019; Galloway et al., 2020). The pixie robber flies’ small bodies and heads could even be to their advantage, as this might be the reason why they are not spotted by their prey when assessing it, particularly when doing so in the shaded and cluttered environments they inhabit (Newkirk, 1963; Martin, 1968; Dennis and Lavigne, 1976). Making sure that the peering movements required for motion parallax go unnoticed by prey could be a crucial condition for using it during predation; the reason why barn owls, known nocturnal predators, also use head movements when assessing prey (Ohayon et al., 2006; Fux and Eilam, 2009) could be that these are hardly visible in darkness. Hunting in shaded environments, however, poses its own sensory challenges. As the number of photons reaching their compound eyes is low, insects living in dark environments tend to have larger lenses and acceptance angles to increase sensitivity. It is not surprising therefore that pixie robber flies do have substantial facet lens diameters (*D*_f_=68.6 µm) in their fovea, almost twice as wide as those of damselflies (34.2 µm; Scales and Butler, 2016), contributing to a relatively small *F*-number of 1.39. However, this low value is also likely to be a consequence of the spatial constraints imposed by head size on facet diameter and ommatidium length (Stavenga, 2003). Interestingly, pixie robber flies have very thin rhabdomeres with diameters of *D*_rh_=0.78 µm (averaged between the major and minor axes), approaching the functional limit of fly photoreceptors, calculated to be 0.7 µm (Horridge et al., 1976). This produces a very small acceptance angle, which we calculate optically as Δρ=0.47 deg, about half the size of these robber flies' interommatidial angle (Δϕ=0.84 deg). If confirmed physiologically, this would imply severe image undersampling in favour of resolution. This is an unusual trade-off for compound eyes operating at low light levels (Snyder, 1977; Snyder et al., 1977; Horridge, 1978; Land, 1997), but one that has been found in other small insects with a predatory lifestyle (Horridge and Duelli, 1979). Some of the negative effects of having such a small acceptance angle could be partially mitigated by the pixie robber fly's low *F*-number, though these small predators could present additional interesting trade-offs in photoreceptor physiology and connectivity. This might even explain why robber flies assessed prey more slowly and for longer compared with damselflies, as this could allow them to gather visual information over time and overcome the low signal-to-noise ratio implied by their very thin photoreceptors. It is worth noting, however, that these considerations are only true for the foveal ommatidia considered in this paper, and that a different trade-off might be present for the rest of the compound eye.

Three-dimensional estimations of prey size and/or distance during predatory behaviour have long been thought to rely on the binocularity of animals with large eyes and/or high visual acuity (Collett, 1977; Fox et al., 1977; Rossel, 1983; Nityananda and Read, 2017; Wardill et al., 2017; Supple et al., 2020). However, this and other examples (Gilbert, 1997; Kral et al., 2000; Gonzalez-Bellido et al., 2011) show that small predators with limited visual systems can still successfully meet the same visual challenges as larger competitors, although with small but acceptable sacrifices.

## Supplementary Material

10.1242/jexbio.251710_sup1 Supplementary information
