# Supplementary material for: Predation via motion parallax in one of two gleaning insects
Source: J Exp Biol. 2026 Mar 18;229(6):jeb251710. doi: 10.1242/jeb.251710 (PMC13035275; doi:10.1242/jeb.251710)
Supplement: Supplementary information [file jexbio-229-251710-s1.pdf]

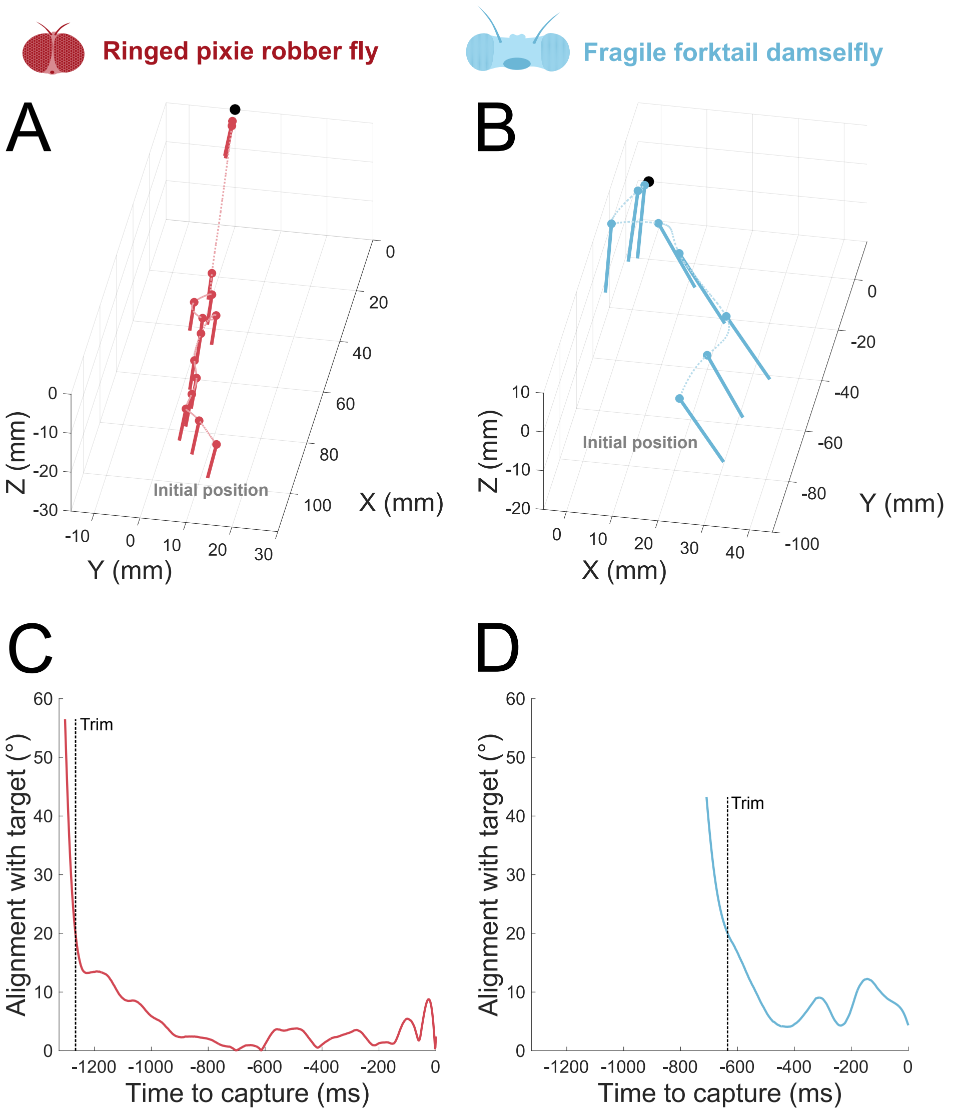

Fig. S1. Predator body angle when assessing and attacking prey. A) The trajectory of a pixie robber fly (dotted line) assessing and attacking a fruit fly. The position of the fly every 100 ms is shown as a point (thorax) and line (abdomen). The target position is represented as a black point. B) The trajectory of a damselfly assessing and attacking a fruit fly. Details are as in panel A. C) The body alignment with the target over time of the robber fly's assessment and attack shown in A. The vertical line represents the timepoint when the fly's body aligns under 20° with the target, which was used to trim attacks. D) The body alignment with the target over time of the damselfly's assessment and attack shown in B. Details are as in panel C.

Journal of Experimental Biology • Supplementary information

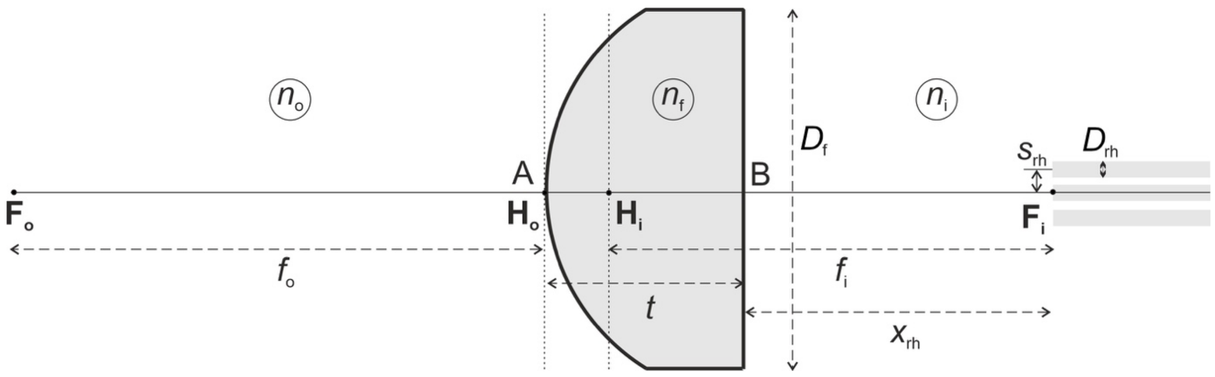

Fig. S2. Geometrical optics diagram of a pixie robber fly facet lens and underlying rhabdomeres. The lens front surface is strongly curved, the back surface is flat. D_f: lens diameter; t: thickness; n_o, n_f, and n_i: refractive indices of object space, facet lens, and image space; F_o and F_i: focal points of object and image space; f_o and f_i: focal lengths of object and image space; A and B: axial lens points; x_{rh}: back focal distance; D_{rh}: diameter of rhabdomere; s_{rh}: distance between rhabdomere axes.

Journal of Experimental Biology • Supplementary information

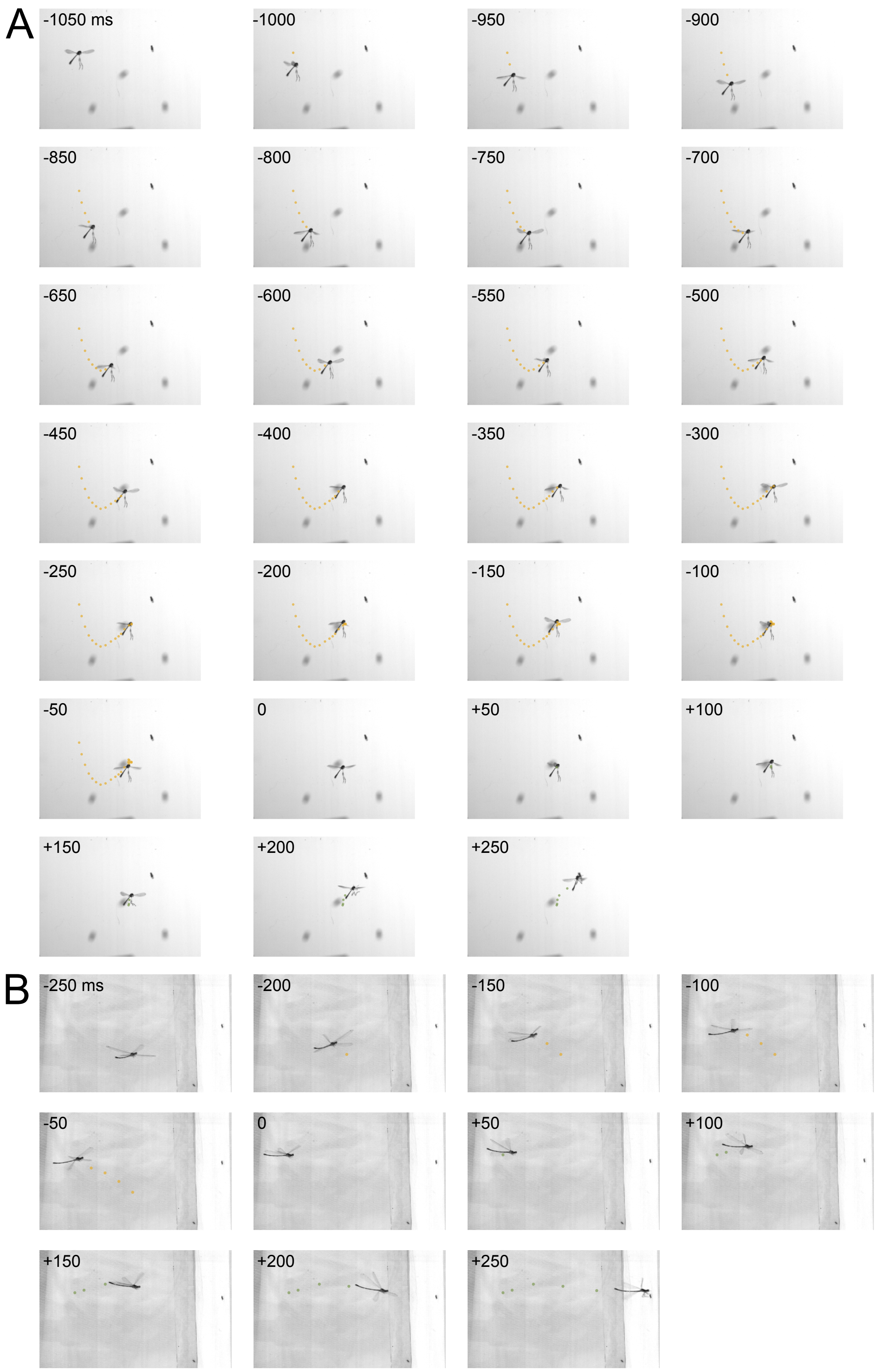

Fig. S3. Sequential images of pixie robber flies and damselflies gleaning static prey. Thorax positions of previous images in the sequence have been indicated as dots and colour coded for behavioural phase (yellow for assessments, green for attacks).

Journal of Experimental Biology • Supplementary information

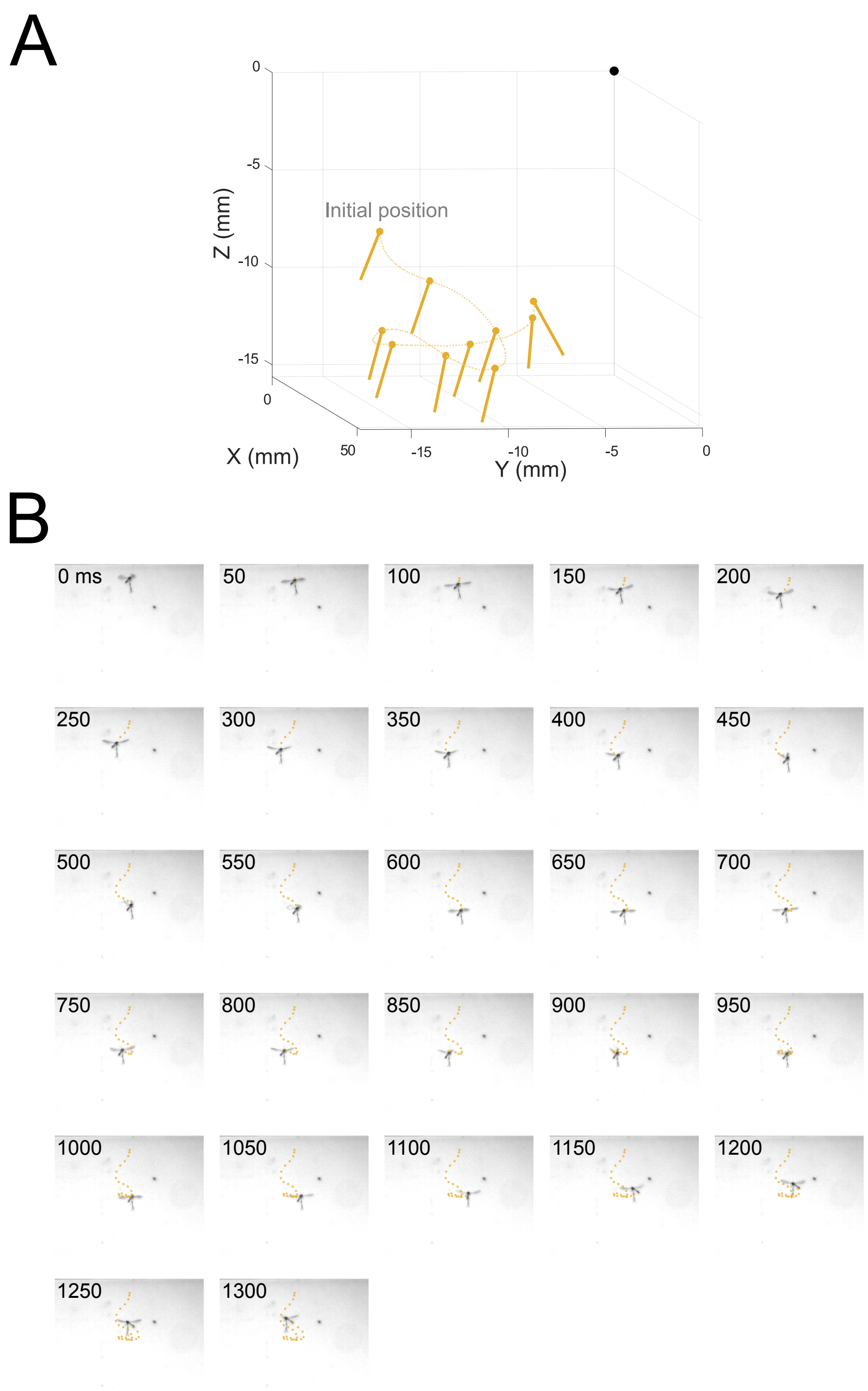

Fig. S4. Pixie robber flies can assess prey without performing an attack. A) The trajectory of a pixie robber fly (dotted line) assessing a static target without surging. The position of the fly every 100 ms is shown as a point (thorax) and line (abdomen). The target position is represented as a black dot. B) Sequential images of a pixie robber flies assessing, but not attacking, unsuitable prey. Thorax positions of previous images in the sequence have been indicated as dots.

Journal of Experimental Biology • Supplementary information

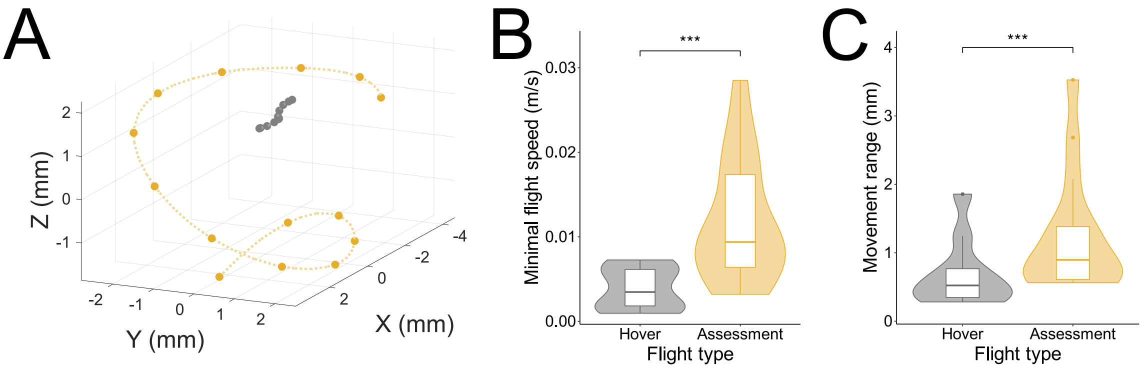

Fig. S5. Pixie robber flies are capable of flying at lower speed and with less translational movements than during assessments. A) The trajectory of a pixie robber fly during an assessment (yellow dotted line), compared to a hovering flight (grey dotted line). The position of the thorax is shown every 30 ms (yellow and grey points). B) The minimal flight speed of hovering flights (grey) and assessments (yellow). C) The movement range of hovering flights (grey) and assessments (yellow). Significance values: ***=p<0.001. Sample size: n=10 flights.

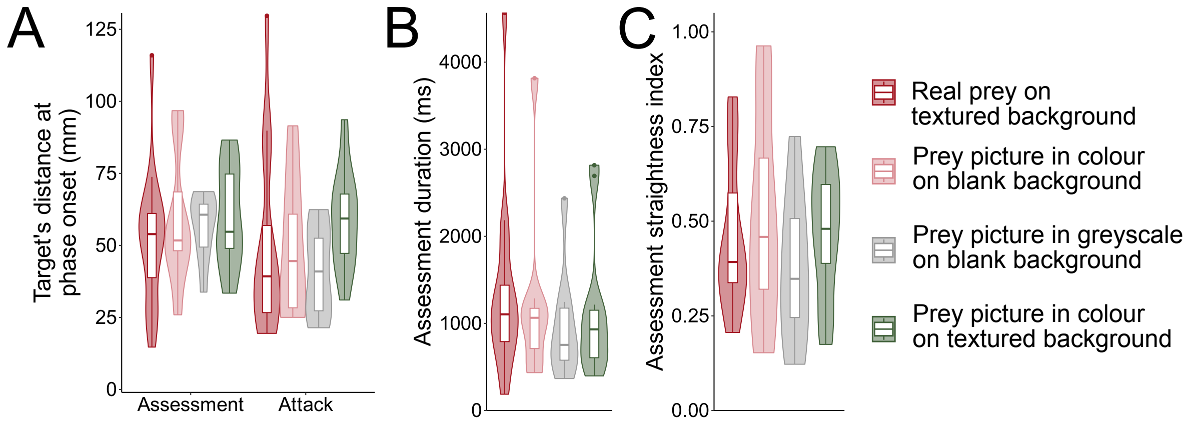

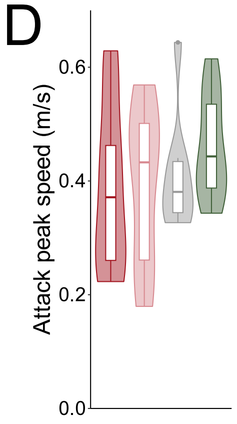

E

	Target's distance at assessment onset	Assessment duration	Assessment straightness index	Target's distance at attack onset	Attack peak speed
**Real prey on textured background** *vs* **prey picture in colour on blank background**	$t(4.73) = 1.62$<br>$p = 0.169$	$W = 10$<br>$p = 0.368$	$W = 20$<br>$p = 0.570$	$W = 25$<br>$p = 0.154$	$t(9.19) = 0.163$<br>$p = 0.874$
**Prey picture in colour on blank background** *vs* **prey picture in colour on textured background**	$t(6.31) = 1.80$<br>$p = 0.119$	$W = 12$<br>$p > 0.999$	$t(3.83) = 0.116$<br>$p = 0.913$	$t(5.76) = 5.12$<br>$p = 0.002$<br>**	$t(7.81) = 0.411$<br>$p = 0.692$
**Prey picture in colour on blank background** *vs* **prey picture in greyscale on blank background**	$t(4.11) = 1.41$<br>$p = 0.231$	$W = 7$<br>$p = 0.886$	$t(3.86) = 1.59$<br>$p = 0.189$	$t(5.34) = 2.00$<br>$p = 0.098$	$W = 1$<br>$p = 0.057$

Fig. S6. Pixie robber flies behave similarly towards real prey, prey pictures, prey in greyscale, and prey on textured backgrounds. A) The target distance at the initiation of the assessment and attack for each stimulus used. B) The duration of the assessment for each stimulus used. C) The straightness index of the assessment for each stimulus used. D) The peak speed of the attack for each stimulus used. E) A summary of the statistical tests comparing attacks to real fruit flies and fruit fly pictures, virtual prey on blank and textured backgrounds, and virtual prey in colour and greyscale. Sample size: n=16 for real prey on textured background, n=8 for prey picture in colour on blank background, n=8 prey picture in greyscale on blank background, n=12 prey picture in colour on textured background.

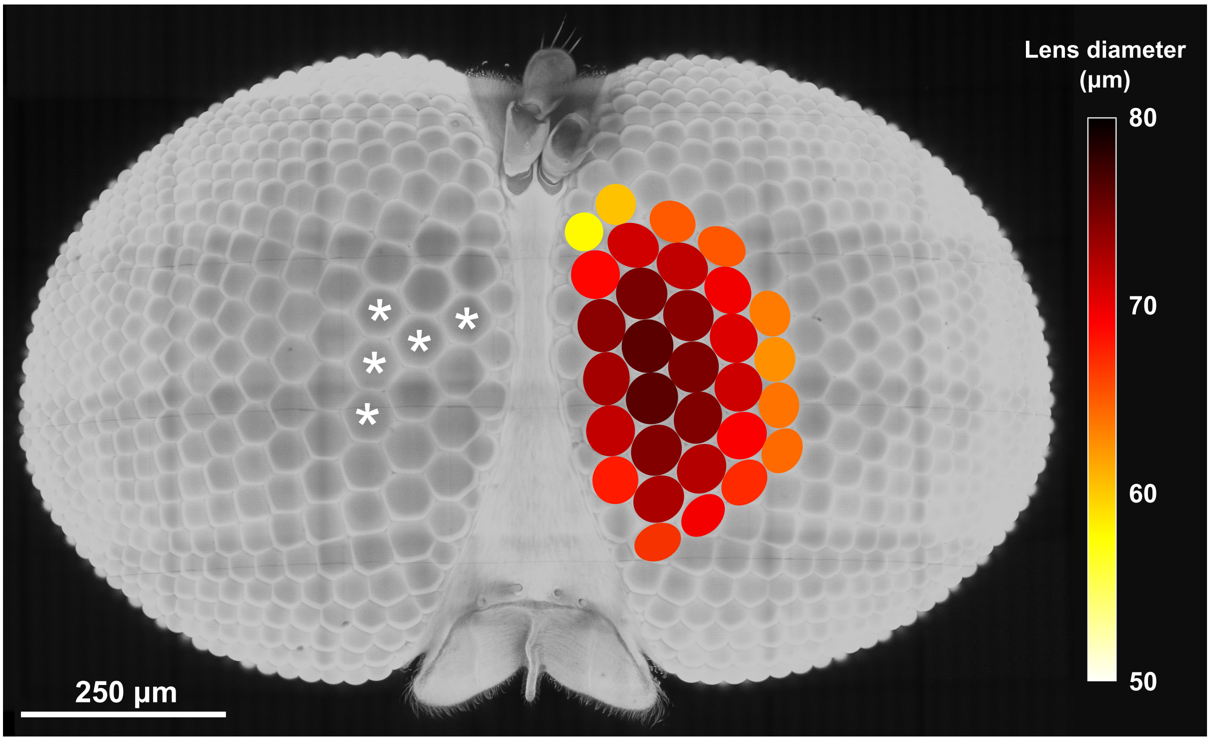

Fig. S7. A two-photon scan shows the acute zone on the eye of a pixie robber flies, where transmission electron micrographs were centred. The lenses of the pixie robber fly's left eye are colour coded for their size (lighter colour indicate smaller lenses). On the right eye, asterisks indicate the locations where sections were centred for acquisition and measurement of electron micrographs.

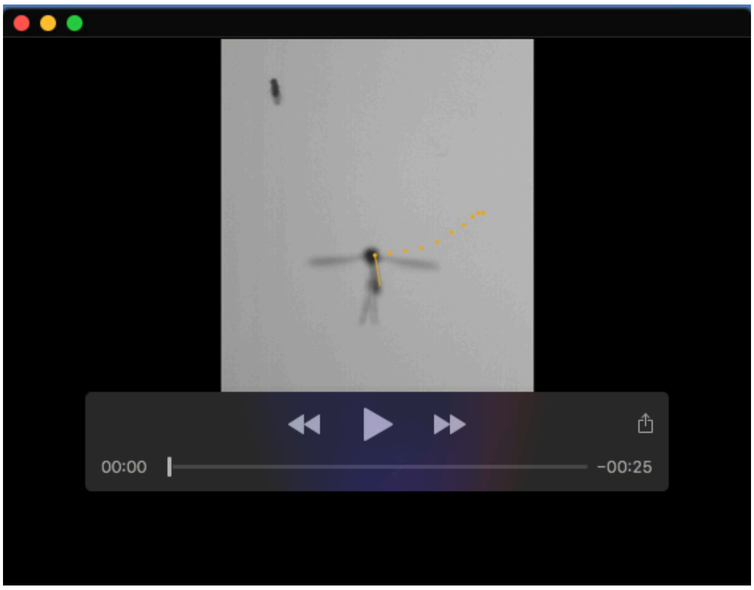

Movie 1. Hunting sequences of pixie robber flies and damselflies to real fruit flies. The digitised positions of the thorax (point) and abdomen (line) are overimposed to the current frame every 40 ms and only past positions of the thorax are kept for the entire sequence. The thorax and abdomen are colour-coded according to the behavioural phase classification, *assessment* (yellow) or *attack* (green). Videos were recorded at 1000 frames per second and shown at 50 frames per second.

Journal of Experimental Biology • Supplementary information

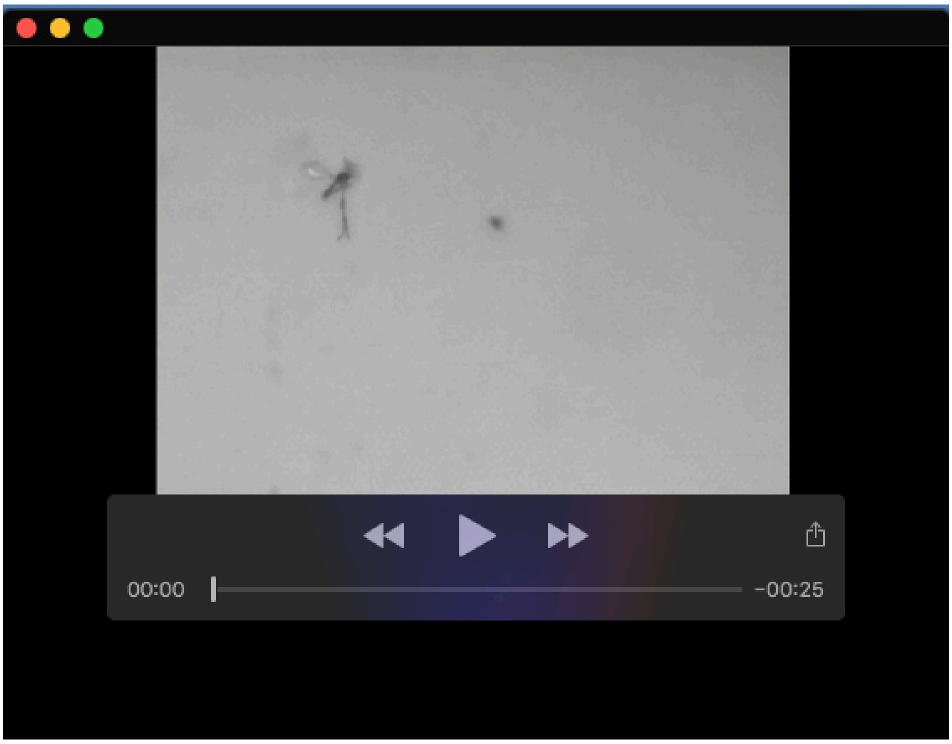

Movie 2. A pixie robber fly assessing prey without performing an attack. The video was recorded at 1000 frames per second and shown at 100 frames per second.

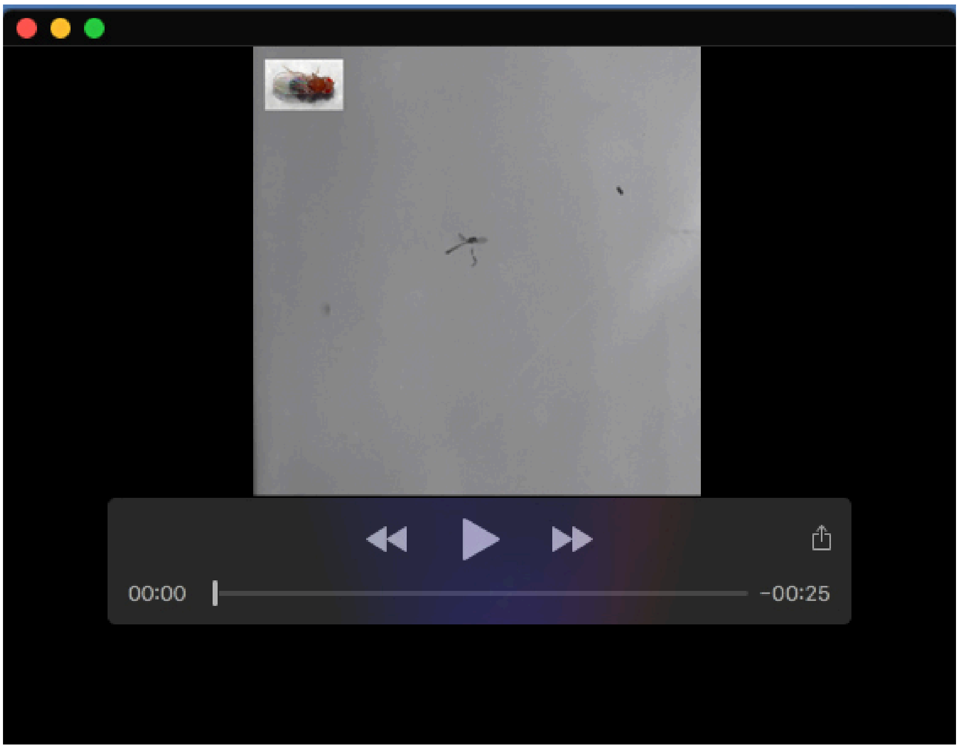

Movie 3. Pixie robber flies attacking prey images of different shapes and colour. The stimuli used are shown magnified and in full colour in the top left corner for each behavioural sequence. Videos were recorded in greyscale at 1000 frames per second and shown at 100 frames per second.

Supplementary Materials and Methods

Consider a thick lens (**Fig. S2**), with thickness t, which images objects in object space (o) into image (i) space. The curvature of the front surface is r_o and that of the back surface r_i. If the refractive indices of object space, facet lens, and image space are n_o, n_f, and n_i, respectively, the facet lens power is

$$P_f = P_o + P_i - \frac{t}{n_f} P_o P_i$$

where the powers of the front and back surfaces are

$$P_o = \frac{n_f - n_o}{r_o} \quad \text{and} \quad P_i = \frac{n_i - n_f}{r_i}$$

A thick lens has two principal planes, given by the axial principal points H_o and H Their distances to the axial lens points A and B are given by

$$AH_o = \frac{n_o}{P_f}\frac{t}{n_f} P_i \quad \text{and} \quad BH_i = -\frac{n_i}{P_f}\frac{t}{n_f} P_o$$

The focal lengths of object and image space are related to the lens power by

$$f_o = \frac{n_o}{P_f} = \frac{n_o}{n_f - n_o} r_o \quad \text{and} \quad f_i = \frac{n_i}{P_f} = \frac{n_i}{n_f - n_o} r_o$$

As the back surface of the robber fly facet lenses is about flat, it follows that P_i=0 and hence $P_f = P_o$, and furthermore that AH_o=0 and BH_i=-n_i t/n_f. Accordingly, the back focal distance, i.e., distance of the facet lens' back surface to the image focal plane, where the rabdomere tips are expected, is

$$x_{rh} = f_i - \frac{n_i}{n_f} t = \frac{n_i}{n_f - n_o} r_o - \frac{n_i}{n_f} t$$

The refractive index values are taken to be n_o=1.00, n_f=1.43, and n_i=1.34. With the front surface curvature r_o=41.2 µm, the focal distance (in air) f_o=95.8 µm, and thus with the facet lens diameter D_f=69.9 µm, the F-number is $F=f_o/D_f$=1.37. With the thickness t=31.6 µm, x_{rh}=97.8 µm.

Journal of Experimental Biology • Supplementary information
